# Antimicrobial Activity of Two Garlic Species (*Allium Sativum* and *A. Tuberosum*) Against Staphylococci Infection. *In Vivo* Study in Rats

**DOI:** 10.15171/apb.2017.015

**Published:** 2017-04-13

**Authors:** Paulo César Venâncio, Sidney Raimundo Figueroba, Bruno Dias Nani, Luiz Eduardo Nunes Ferreira, Bruno Vilela Muniz, Fernando de Sá Del Fiol, Adilson Sartoratto, Edvaldo Antonio Ribeiro Rosa, Francisco Carlos Groppo

**Affiliations:** ^1^ Department of Exact Sciences, Technical School of Limeira, Cotil, UNICAMP, Limeira, São Paulo, Brazil.; ^2^ Department of Physiological Sciences, Piracicaba Dental School, UNICAMP, Piracicaba, São Paulo, Brazil.; ^3^ Department of Pharmacological Sciences, School of Pharmacy of Sorocaba, UNISO, Sorocaba São Paulo, Brazil.; ^4^ Research Center for Chemistry, Biology and Agriculture, CPQBA, UNICAMP, Paulínia, São Paulo, Brazil.; ^5^ Xenobiotics Research Unit, PUC-Paraná, Curitiba, Paraná, Brazil.

**Keywords:** Garlic, Chinese chive, Amoxicillin, Staphylococcus aureus, Infection

## Abstract

***Purpose:*** This study observed the effect of garlic extracts and amoxicillin against an induced staphylococcal infection model. MIC and MBC were also obtained for aqueous extracts of Allium sativum (Asa) and Allium tuberosum (Atu) against Staphylococcus aureus penicillin-sensitive (PSSA - ATCC 25923) and MRSA (ATCC 33592).

***Methods:*** Granulation tissues were induced in the back of 205 rats. After 14 days, 0.5 mL of 10^8^ CFU/mL of PSSA or MRSA were injected inside tissues. After 24h, animals were divided: G1 (Control) – 0.5 mL of NaCl 0.9%; G2 – Asa 100 mg/kg or 400mg/kg; G3 – Atu 100 mg/kg or 400 mg/kg; G4 – amoxicillin suspension 50 mg/kg, considering PSSA infection; and G5 (Control) - 0.5 mL of NaCl 0.9%; G6 – Asa 400mg/kg; G7 – amoxicillin 50 mg/kg; and G8 - Asa 400 mg/kg + amoxicillin 50 mg/kg for MRSA. All treatments were administered P.O. every 6h. Animals were killed at 0, 6, 12 and 24h. Samples were spread on salt-mannitol agar. Colonies were counted after 18 h at 37 °C. Atu was not able to inhibit or kill PSSA and MRSA. Considering Asa, MIC and MBC against PSSA were 2 mg/mL and 4 mg/mL, respectively; and 16 mg/mL and 64 mg/mL against MRSA.

***Results:*** No effect was observed in vivo for control, Asa 100 mg/kg and Atu 100 mg/kg, while amoxicillin, Atu 400 mg/kg and Asa 400 mg/kg decreased PSSA counts in all-time points. No effect of any group against MRSA was observed at any time.

***Conclusion:*** Thus, A. sativum and A. tuberosum were able to reduce PSSA infection, but not MRSA infection.

## Introduction

Among all staphylococci,* Staphylococcus aureus* is the most important cause of infectious diseases in humans. This microorganism is part of the commensal microbiota, causing opportunistic infections under appropriate conditions.^1^ Besides, intrinsic virulence factors, life-threatening staphylococcal infections occur due to its ability to develop antibiotic resistance.^2^ The antimicrobial resistance against penicillins is remarkable in the most known *S. aureus* strains. The minimum inhibitory concentration (MIC) of amoxicillin against penicillin-sensitive *S. aureus* strains is usually below to 0.5 µg/mL.^3^ The beta-lactams’ MIC against resistant strains could be hundred times higher than the usual MIC of sensitive strains. Thus, there is a clear need for novel antimicrobials or new methods to improve the efficacy of old antibiotics.

Natural products are the most important source of innovative contributions to antimicrobial therapeutic.^4^ The genus *Allium* is often used and studied in medicine as antibacterial and anti-viral agents among many other properties.^5^ There are over 600 species of *Allium* found throughout the world, most of them in Asia. Most species have characteristic aroma and odor; some are used as ornamentals or spices. Garlic is often used in many forms (oils, powder, etc.), being also present in many pre-packaged foods, soups and bread used in a daily diet.^6^

Among the species of *Allium spp*., *A. tuberosum* ("Chinese chive") is an important ingredient in Asian cuisine, being used as a medicinal herb for many disorders and diseases.^7^ Both pressed juice and essential oil showed inhibition properties against a broad range of gram-positive and gram-negative microorganisms as well as many fungi.^8^

*Allium sativum* contains several biologically active chemical constituents, such as alliin, allicin, alliinase, S-allylcysteine ​​and other organosulfur compounds.^9^ Alliin is a natural sulfoxide constituent of fresh garlic. It is derivate from the amino acid cysteine, being converted to allicin by the enzyme aliinase when bulbs are mashed. Allicin is a precursor of sulphur compounds, responsible for the odor and some of the pharmacological properties of garlic. Once exposed to the atmospheric air, allicin is converted into diallydissulphinate, which has antimicrobial activity.^10,11^

Garlic extract and some of its products, such as diallyl sulphide and diallyl disulphide showed protective action against MRSA infection in mice.^12^ The additive or complimentary effect of garlic or its products on some antibiotics against bacterial infections was previously demonstrated *in vitro*^13^ and *in vivo*.^14^

The aim of this study was to observe the effect of garlic extracts alone or in combination with amoxicillin on the staphylococcal infection, which was induced with penicillin-sensitive or penicillin-resistant strains, in rats.

## Materials and Methods

### Preparation of garlic extracts

Garlic bulbs (*Allium sativum*) and Chinese chive (*Allium tuberosum*) were supplied by Research Center for Chemistry, Biology and Agriculture – CPQBA-UNICAMP (Campinas, SP, Brazil). The dry peel around the bulbs was removed and they were used to prepare *A. sativum* extract. *A. tuberosum* extract were prepared using only the plant leaves. Bulbs are the most common part of *A. sativum* used by population and in most of studies. Considering *A. tuberosum* the few studies in literature used its leaves as a primary source for extracts. Both aqueous extracts were prepared by using 100 g of fresh rootless bulbs of *A. sativum* or 100 g of *A. tuberosum* leaves and 100 mL of distilled/deionized water. Both plants were ground in a blender for 10 min and the resulting extracts were filtered in qualitative paper filters, and sterilized through 0.2 µm membrane filters by using a vacuum pump. All residues were weighed, and the concentration of the final solution of both extracts was considered 25% (w/v) or 250 mg/mL, as previously described.^15,16^

### Analysis of extracts compounds

The extracts were analyzed using a gas chromatograph (Hewlett-Packard – HP-5890) with a mass-selective detector (Hewlett-Packard – HP-5971), equipped with DB-1 capillary column (25 m x 0.2 mm x 0.33 µm). Carrier gas was helium at a flow rate of 1.0 mL/min. The injection temperature was 240 °C and the detector was set at 300 °C. The column temperature program was 60 °C (2 min) to 300 °C at 4 °C/min.

The injection volume was 1.0 µL and the detector was a quadropole system with ionization energy of 70 eV. Both extracts were assayed under the same conditions, using the NIST98 electronic library for GC/MS.

### Bacteria

Both *Staphylococcus aureus* ATCC 25923 (penicillin sensitive - PSSA) and ATCC 33592 (methicillin resistant - MRSA) were used in the *in vitro* and *in vivo* models. Both strains were kept in TSA medium.

### In vitro study

#### Minimal inhibitory (MIC) and bactericidal (MBC) concentrations

Eleven culture tubes containing 5 mL of Mueller–Hinton broth were used for each strain. Two-fold progressive concentrations of both extracts were added in the first 10 tubes. The last tube had microorganisms only (positive control). An extra tube was used as negative control (without microorganisms or extracts). The extracts’ concentrations were 1, 2, 4, 8, 16, 32, 64, 128, 256 and 512 mg/mL. Amoxicillin was used in concentrations of 0.5, 1, 2, 4, 8, 16, 32, 64, 128 and 256 µg/mL for PSSA and 32, 64, 128 and 256, 512, 1024, 2048, 4096 and 8192 µg/mL for MRSA.

All tubes received 250 µL of 10^6^ CFU/mL of either PSSA or MRSA. Tubes were incubated in aerobic conditions at 37 °C. Bacterial concentration was adjusted by using a spectrophotometer. After 18 h, the culture medium was observed and the turbidity of medium was measured and compared with the negative-control tubes. The first tube without bacterial growth was considered as MIC. Amounts of 5 µL from the tubes without bacterial growth were spread on Petri dishes containing BHI agar. The first concentration without any bacterial growth on agar was considered as MBC.

#### In vivo study

This study was approved by the Ethical Committee of Animal Experimentation of Biology Institute-UNICAMP (protocol # 1457-1).

Two assays were carried out. The first one was designed to compare both garlic extracts against the PSSA infection. The second one was designed to compare the effect of *A. sativum*, which was the most efficient formulation in assay 1, and amoxicillin against MRSA infection.

#### First assay

140 adult-male Wistar rats (*Rattus norvegicus*-albinus), 60 days of age and weighing 175 g ± 25 g, were obtained from CEMIB-UNICAMP (Centro de Bioterismo – ICLAS Monitoring/Reference Centre, Campinas, Brazil).

Granulation tissue was induced by insertion of four sterilized polyurethane sponge discs (density = 35 kg/m^3^) subcutaneously in the back of each animal, according to a method previously described.^17^ These sponge discs (Proespuma Com. & Ind. Ltd., Sao Paulo, Brazil) were 12 mm in diameter and 5 mm thick, weighing 12 ± 1 mg, being all of them were previously sterilized.

Animals were anesthetized by an injection of ketamine (40-87 mg/kg/I.P.) and xylasine (5-13 mg/kg/I.P.). After careful antisepsis with povidone-iodine, the back skin was incised and the sponge discs were carefully distributed considering a distance of approximately 2 cm among them, two in tail direction and two in head direction. Meperidine chlorhydrate (2 mg/kg/I.M.) was used to control post-operative pain. After 14 days of sponge insertion, a well-delimited granulation tissue was formed around and inside the sponge disks. This tissue was used as a scaffold for the staphylococcal infection.

In order to establish the staphylococcal infection, a careful antisepsis was carried out in the back of all animals and 0.5 mL of a suspension of PSSA (10^8^ CFU/mL) was injected into the four granulation tissues. After 24 hours of infection, the animals were submitted to the following treatments:

1. Control – 20 animals received 0.5 mL of 0.9% NaCl. These animals were killed (n = 5 per group) right after the administration (t=0) and after 6, 12 and 24 hours after saline administration.
2. *Allium sativum* - 15 animals received 100 mg/kg of an *A. sativum* aqueous extract and 15 animals received 400 mg/kg of an *A. sativum* aqueous extract.
3. *Allium tuberosum* – 15 animals received 100 mg/kg of an *A. tuberosum* aqueous extract and 15 animals received 400 mg/kg of an *A. tuberosum* aqueous extract.
4. Amoxicillin – 15 animals received amoxicillin suspension 50 mg/kg/P.O. every 6 hours.

Treatments were administered P.O. every 6 hours. Animals of groups 2, 3, and 4 were killed after 6, 12 and 24 hours after drugs administration.

#### Second assay

After 14 days of sponge implants and careful antisepsis, 65 animals received 0.5 mL of a suspension of 10^8^ CFU/mL MRSA strain (*S. aureus* ATCC 33592) into the granulation tissue. After 24 hours of the infection procedure, the animals were divided into the following groups:

1. Control – 20 animals received 0.5 mL of NaCl 0.9% P.O. every 6 hours. These animals were killed (n = 5 per group) right after the administration (t = 0) and after 6, 12 and 24 hours after saline administration.
2. *Allium sativum* - 15 animals received *A. sativum* aqueous extract 400 mg/kg. Both extracts were administered P.O. every 6 hours and the animals were killed after 6, 12 and 24 hours after the first administration.
3. Amoxicillin– 15 animals received amoxicillin suspension 50 mg/kg/P.O. every 6 hours. These animals were killed after 6, 12 and 24 hours after amoxicillin administration.
4. *A. sativum* + amoxicillin - 15 animals received both *A. sativum* extract 400 mg/kg and amoxicillin suspension 50 mg/kg. Both extract and amoxicillin suspension were administered P.O. every 6 hours. Animals were killed after 6, 12 and 24 hours after the first administration.

#### Granulation tissue removal

All infected granulation tissues were surgically removed after general anesthesia and blood removal by cutting carotid plexus. Tissue samples were individually disposed into assay tubes with 10 mL of 0.9% NaCl. Tubes were weighed before and after the tissue insertion. Samples were dispersed using an ultrasonic system (Vibra Cell 400W, Sonics & Materials Inc, Danbury, CT, USA) and 10 µL of the resulting suspension was spread on salt mannitol agar and incubated at 37 °C. Eighteen hours after incubation, the colonies were counted by using a manual colony counter.

#### Statistical analysis

Results were submitted to the Bartlett’s test to assess variance homogeneity, and the Kolmogorov & Smirnov test to observe data distribution. Data of the first assay were transformed and analyzed by two-way ANOVA and Tukey’s test adjusted for multiple comparisons (*post hoc*). Second assay data were submitted to Kruskal-Wallis’ and Dunn’s tests. GraphPad Prism 6.0 was used to analyze all data, considering 5% of a significant level.

## Results

### Gas chromatography profile

**Figure 1:**
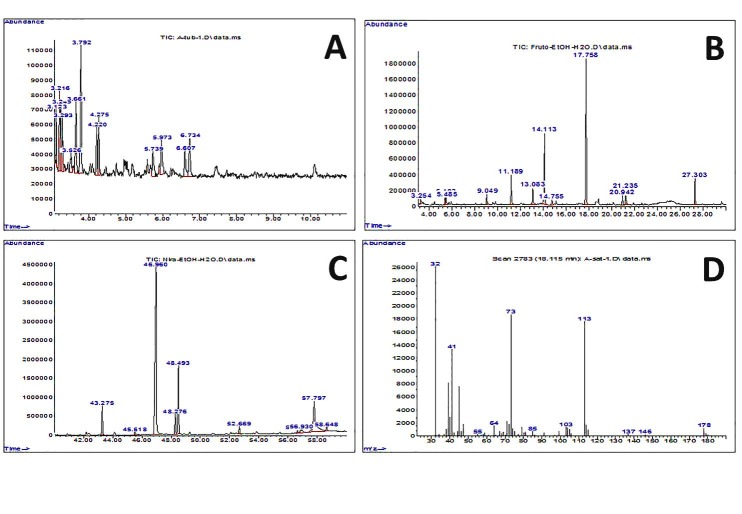
GC-Chromatograms of both *A. tuberosum* (A and C) and *A. sativum* (B and D) extracts. Aqueous extracts are presented in A and B and the hydroalcoholic ones in C and D.

Figure 1 and (Table 1) show respectively the gas chromatography profile and the probable chemical composition of both *A. tuberosum* and *A. sativum* aqueous and hydroalcoholic extracts.

The most abundant compound of the *A. tuberosum* aqueous extract was not identified by gas chromatography, being only 8% of its compounds identified. However, *A. tuberosum* hydroalcoholic extract had phytol as the most abundant compound (48.4%) followed by other acyclic compounds (23.4%) and only 1.8% of sulphides. *A. sativum* aqueous extract showed 82% of sulphur compounds, being disulphides the most prevalent ones. The hydroalcoholic extract of *A. sativum* showed trisulphides (31%) as the most abundant compound.

### MIC and MBC

*A. tuberosum* was not able to inhibit or kill both resistant and sensitive staphylococci strains in MIC and MBC tests. *A. sativum* and amoxicillin inhibited (MIC) the penicillin-sensitive strain respectively at 2 mg/mL and 0.1 µg/mL. They killed (MBC) this strain at 4 mg/mL and 2 µg/mL, respectively. However, it was necessary 2048 µg/mL of amoxicillin to inhibit the MRSA strain and 8192 µg/mL to kill it. *A. sativum* was able to inhibit MRSA at 16 mg/mL, being able to kill it at 64 mg/mL.

**Table 1:** Probable chemical composition of both aqueous and hydroalcoholic extracts of *A. tuberosum* and *A. sativum*.

	***Allium***	**Retention time (min)**	**Kovats index**	**Probable identification**	**Class**	**Relative %**
***Aqueous extracts***	*tuberosum*	3.22	---	2,5-dimethyl-heptane	Alkane	1.0
		3.25	---	ethylcyclohexane	Alkane	0.7
		3.61	---	1-propene,3,3’-thiobis or diallyl sulfide	**Monosulfides**	0.6
		3.79	---	p-xylene	Aromatic	2.3
		4.23	---	o-xylene	Aromatic	0.8
		4.28	900	nonane	Alkane	1.0
		6.61	994	1,2,4-trimethylbenzene	Aromatic	0.8
		6.74	999	decane	Alkane	1.0
	*sativum*	3.21	---	2,5-dimethyl-heptane	Alkane	1.0
		3.77	---	p-xylene	Aromatic	2.3
		4.26	900	nonane	Alkane	1.0
		6.61	994	1,2,4-trimethylbenzene	Aromatic	0.8
		13.45	1183	3-vinyl-1,2-dithiacyclohex-4-ene	**Disulfides**	21.7
		14.50	1209	3-vinyl-1,2-dithiacyclohex-5-ene	**Disulfides**	57.3
		18.12	1296	di-2-propenyl trisulfide	**Trisulfides**	3.0
***Hydroalcoholic extracts***	*tuberosum*	5.73	958	Dimethyl trisulfide	**Trisulfides**	0.5
		8.55	1053	S-methyl methanethiosulfonate	**Disulfides**	1.3
		43.28	1981	palmitic acid ethyl ester	Ester	5.2
		46.96	---	phytol	Alcohol	48.4
		48.27	---	linoleic acid ethyl ester	Ester	3.7
		48.50	---	linolenic acid ethyl ester	Ester	13.0
		52.67	---	ethyl 3-(4-hydroxy-3-methoxyphenyl)propanoate	Ester	1.4
	*sativum*	13.08	1174	3-vinyl-1,2-dithiacyclohex-4-ene	**Disulfides**	3.5
		14.11	1200	3-vinyl-1,2-dithiacyclohex-5-ene	**Disulfides**	13.9
		17.76	1288	di-2-propenyl trisulfide or diallyl trisulfide	**Trisulfides**	31.2
		27.30	1523	di-2-propenyl tetrasulfide or diallyl tetrasulfide	**Tetrasulfides**	5.1
		42.20	1948	dibutyl-o-phthalate	Ester	2.2
		48.26	---	linoleic acid ethyl ester	Ester	1.8

### First in vivo assay

**Figure 2:**
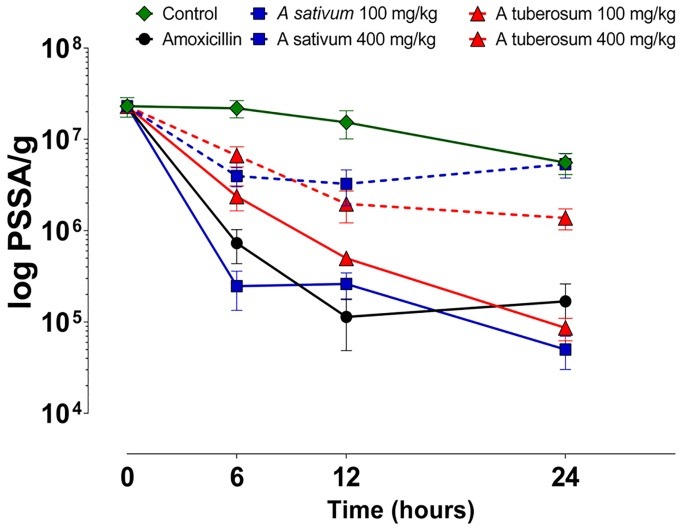
Effect of the treatments on the concentration (mean±SD) of PSSA per gram of tissue in all-time points.

Figure 2 shows the effect of treatments in the number of bacteria in all-time points. There are no significant differences (p>0.05) among periods for control, *A. sativum* 100 mg/kg and *A. tuberosum* 100 mg/kg. Amoxicillin decreased the CFU counts after 6 and 12 hours, but no differences were found between 24 hours and 6 or 12 hours. *A. tuberosum* 400 mg/kg decreased the bacterial counts starting from 6 to 24 hours and *A. sativum* showed a decrease after 6 and 24 hours.

Comparison among groups in each period showed no significant differences among control, *A. sativum* 100 mg/kg and *A. tuberosum* 100 mg/kg, except for 6 hours period, when both aqueous solutions significantly decreased the CFU in comparison with control. Amoxicillin and *A. sativum* 400 mg/kg decreased CFU in comparison with control, *A. sativum* 100 mg/kg and *A. tuberosum* 100 mg/kg in each period, but they did not differ from *A. tuberosum* 400 mg/kg. This formulation also decreases CFU in comparison with control, *A. sativum* 100 mg/kg and *A. tuberosum* 100 mg/kg in all periods, except for 6 hours.

### Second in vivo assay

**Figure 3:**
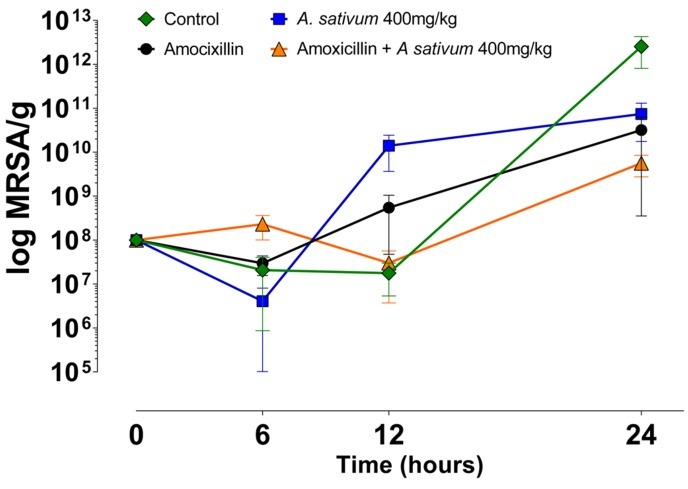
Effect of the treatments on the concentration (mean±SD) of MRSA per gram of tissue in all-time points.

Figure 3 shows the effect of treatments against the resistant bacteria in all-time points. CFU increased after 12 h for control and amoxicillin + *A. sativum*, and after 6 hours for *A. sativum* 400 mg/kg and amoxicillin. Comparison among groups in the 6 hours period showed greater number of CFU for the amoxicillin group than the others groups, which did not differ one from each other. After 12 hours, control showed bigger number of CFU than amoxicillin alone or combined with *A. sativum*. No significant differences were observed in this period among *A. sativum* and the other groups. No significant differences were observed at the 24 hours period.

## Discussion

Among many other models, the use of PVC or polyurethane sponges to induce a well-delimited infection was previously described for research on anti-staphylococci substances.This model allowed a well-delimited staphylococcal infection development, since one of the most important phenomena for the infection establishment is bacterial surface adherence. The validity of the model was previously demonstrated for acute staphylococcal infection.^17^

Most of the important infections results from bacterial adherence on some tissue surface, and the first line of the host defense against the bacterial invasion also request a surface to exert its defensive function.^18^ Thus, the surface provided by the granulomatous tissue was suitable to both staphylococcal infection development and host defensive events.

It is remarkable that amoxicillin was not able to kill all the PSSA microorganisms, and, thus, it did not eradicate the staphylococcal infection. This result is similar to the ones previously obtained using the same model. The main reason for this phenomenon is probably due to the penicillin mechanism of action. It is well stablished that bacteria growing in tissues surrounded by immune cells show slow reproduction, which decreases the penicillin activity.^17^

As anticipated, the chemical composition differed between the two garlic species. The main chemical composition of *A. tuberosum* is thiosulphinates,^19^ and various steroidal saponins,^20^ which were observed in both *A. tuberosum* hydroalcoholic and aqueous extracts in the present study.

*A. sativum* chemical composition was similar to the one previously described by others.^9,21,22^ Discrepancies on chemical composition of *A. sativum* can occur due to the method to obtain the plant extract. In the present study, *A. sativum* extract was obtained as previously described,^15,23^ and it provided antimicrobial activity in both *in vivo* and *in vitro* assays. In addition, we used raw garlic extracts since these extracts showed higher antimicrobial activity than some isolated compounds, such as allicin.^12,24^ Besides, progressive concentrations of a raw extract of *A. sativum* (100 to 1000 µg/mL) strongly inhibited many microorganisms.^25^

The MIC observed for *A. sativum* in the present study (2 mg/mL) against PSSA was lower than MIC observed by,^26^ which observed 11.25 mg/mL against the same strain. Probably, the difference occurred because those authors used agar dilution techniques in spite of liquid medium as used in the present study.

Very few information regarding the antimicrobial activity of *A. tuberosum*, especially against bacteria, was found in literature. The *in vitro* antifungal activity was previously demonstrated for *A. tuberosum* raw extracts against *C. albicans*,^27^ and for the essential oil against Aspergillus species,^8^ observed both antibacterial and antifungal activity of *A. tuberosum* in disk diffusion tests. The aqueous extract was able to inhibit many bacterial species, such as *Bacillus subtilis, Escherichia coli, Pseudomonas aeruginosa,* and* Salmonella typhimurium,* but not *Staphylococcus aureus*.

In the present study, the *in vitro* anti-staphylococcal activity of *A. tuberosum* was not observed against both PSSA and MRSA strains. However, it was able to decrease the PSSA strain counts in the *in vivo* model. Thus, it is possible that the anti-staphylococcal activity of *A. tuberosum* was indirect. As observed in many antimicrobial models, *in vitro* assays do not provide the variability observed in the *in vivo* assays, especially considering immunological aspects.^28^ Differences between *in vitro* and *in vivo* models in antimicrobial research are well recognized and future studies are necessary to address the effects of *A. tuberosum* on host immune response.

*A. sativum* is a well-recognized anti-staphylococcal agent against the biofilm formation in burn wounds,^29^ and against both PSSA and MRSA in an *in vitro* MIC model,^30^ as observed in the present study. In addition, diallyl sulphide and diallyl disulphide from a raw *A. sativum* extract showed protective action against MRSA-infection in both diabetic,^31^ and non-diabetic mice.^12^

Raw garlic extracts (100% and 50% concentrations) administered orally, exhibited comparable effect with both diallyl sulphide and diallyl disulphide, being safer than the two sulphides.^12^ However, in the present study *A. sativum* did not show antibacterial activity against the MRSA strain, despite of the activity against the PSSA strain. The animal model and the garlic concentration used in the present study could have affected the results.

## Conclusion

We concluded that both garlic species showed constitution comparable to other similar strains and they have molecules related to antimicrobial properties. Both species were able to reduce staphylococcal infection, despite only *A. sativum* showed in vitro anti-staphylococci activity. No additive or complementary effect was observed by adding *A. sativum* extract with amoxicillin against the strains studied.

## Acknowledgments

Part of this work was presented at the International Dental Research Association - IADR, Barcelona, Spain, 2010 (poster presentation #1876). This study was supported by FAPESP (foundation for Research Support of the state of São Paulo, grants #2007/08076-5, #08/01021-3).

## Ethical Issues

This study was approved by the Ethical Committee of Animal Experimentation of Biology Institute-UNICAMP (protocol # 1457-1).

## Conflict of Interest

The authors declare no competing interests exist.
